# Illuminating Satellite Cells: Light Sheet Fluorescence Microscopy for 3D Imaging of Murine Skeletal Muscles Damaged by Ex Vivo Forced Eccentric Contraction

**DOI:** 10.1002/jemt.70046

**Published:** 2025-07-14

**Authors:** Rachele Garella, Elisa Imbimbo, Francesco Palmieri, Alessia Tani, Martina Parigi, Flaminia Chellini, Alessandra La Contana, Monica Mattioli Belmonte, Aurora Longhin, Ludovico Silvestri, Chiara Sassoli, Roberta Squecco

**Affiliations:** ^1^ Department of Experimental and Clinical Medicine (DMSC), Section of Physiological Sciences University of Florence Florence Italy; ^2^ European Laboratory for Non‐Linear Spectroscopy (LENS) University of Florence Sesto Fiorentino Italy; ^3^ Department of Experimental and Clinical Medicine (DMSC), Section of Anatomy and Histology, Imaging Platform University of Florence Florence Italy; ^4^ Department of Clinical and Molecular Sciences (DISCLIMO) Marche Polytechnic University Ancona Italy; ^5^ Department of Biomedical and Neuromotor Sciences (DIBINEM) University of Bologna Bologna Italy; ^6^ Department of Physics and Astronomy University of Florence Sesto Fiorentino Italy

**Keywords:** 3D histology, light sheet fluorescence microscopy, muscle repair/regeneration, satellite cells, tissue clearing

## Abstract

In this letter, we put forward the light sheet fluorescence microscopy (LSFM) as a cutting‐edge tool for 3D imaging of whole skeletal muscle, focusing on satellite cells (SCs). SCs represent the resident adult muscle stem cells, normally lying quiescent between the sarcolemma of the myofiber and the surrounding basal lamina. They typically express Pax‐7 and, when activated following damage, they sequentially express specific myogenic regulatory factors including the myogenic determination factor, MyoD, thus starting differentiation towards multinucleated myofibers to repair injured tissue. The present analysis was performed on an ex vivo model of murine skeletal muscle injured by a forced eccentric contraction in isometric condition. The entire muscles were subjected to a tissue clearing and whole‐mount staining process, enabling optical access and specific labeling across the entire intact sample. We performed labeling either with a fluorescent analog of standard hematoxylin and eosin, or with specific immunostaining against Pax‐7 and MyoD. This proof of concept study demonstrates the feasibility of whole‐muscle imaging with LSFM for the evaluation of the spatial arrangement of resting and activated SCs, overcoming the methodological limits of conventional 2D histology. This innovative experimental pipeline can be useful to test novel therapeutic approaches aimed at enhancing tissue regeneration and other biomedical/clinical applications.

## Graphical abstract

This study presents the light sheet fluorescence microscopy (LSFM) as a tool for 3D‐imaging of whole skeletal muscle to reveal satellite cells (SCs), the muscle stem cells that activate following damage to repair injured tissue. After tissue clearing and whole‐mount staining process aimed to enable optical access and specific cell labeling (1), murine EDL muscles both healthy and ex vivo damaged by forced eccentric contraction were observed with LSFM (2) and then subjected to proper image analysis (3) to identify SCs. We have demonstrated the feasibility of using LSFM toanalyse the localization of SCs, imaging the entire skeletal musclein 3D with cellular resolution.

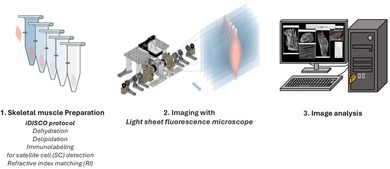

**Summary**

- Light sheet fluorescence microscopy (LSFM) enables 3D visualization of satellite cells (SCs) in the whole skeletal muscle, overcoming 2D histology limitations.
- Tissue clearing enhances dye penetration and optical access, improving SC detection via Pax‐7 and MyoD markers.
- LSFM may represent a novel tool for studying SC activation and muscle regeneration in biomedical research.

## 1 Introduction

Light‐sheet fluorescence microscopy (LSFM) is an advanced technique that enables imaging of whole tissues/organs with high frame rates at cellular resolution. These properties are fundamental to comprehend the three‐dimensional (3D) structure of the tissue of interest and to reveal insights into underlying organ physiology and/or disease including cancer (Liu et al. 2021). In combination with tissue clearing methods, it can be employed to reconstruct the volumetric architecture of biological tissue samples such as brain, heart, and liver (Laurino et al. 2023; Olianti et al. 2022; Silvestri et al. 2021) and can therefore be a useful tool in different clinical applications. Here, we optimized and applied this methodology to perform 3D histology of ex vivo injured skeletal muscles from a mouse model in order to visualize satellite cells (SCs) across the entire sample in 3D. 3D histology using LSFM can have some advantages over standard histology, and this is particularly relevant for investigations of muscle tissue. First, it enables us to visualize the whole muscle structures in three dimensions with minimal tissue distortion and high resolution. Unlike traditional 2D histology, this method preserves spatial relationships between cells and fibers, enabling detailed analysis of muscle architecture, especially useful following muscle damage. Furthermore, LSFM may also enable faster imaging of quite large volumes with less photodamage, making it ideal for capturing dynamic biological processes or rare cellular events in situ. In this regard, SCs represent a small population of resident stem cells of adult skeletal muscle and are regarded as the main actors of skeletal muscle regeneration and post‐natal growth. SCs are mononuclear cells adjacent to myofibers, in particular located between the myofiber sarcolemma and the surrounding basal lamina, hence the name. They express the transcription factor Paired box (Pax)‐7 (von Maltzahn et al. 2013). In healthy muscle, they are quiescent; however, upon a tissue injury or stimulated by growth signals, they activate, proliferating and differentiating to give rise to self‐renewing stem cells and to myogenic progenitors that will fuse together to form new polynucleated myofibers. SCs progress into the myogenic differentiation process by recapitulating essentially the steps of embryonic myogenesis and expressing different myogenic regulatory factors (MRFs) including Myf5, MyoD (myoblast determination protein 1), myogenin, and MRF4, along with Pax‐7, whose sequential expression and hierarchical relationship has been characterized (Guilhot et al. 2024).

Unfortunately, in the case of severe or extended injury with an intense and/or persistent inflammatory reaction or in a pathological microenvironment (e.g., dystrophy), SC functionality can be compromised and overwhelmed by the action of fibroblasts/myofibroblasts producing extracellular matrix components, thus leading to the formation of a non‐functional fibrotic scar which replaces the damaged myofibers (Mahdy 2019; Cardone et al. 2023). The actual challenge in the field of muscle regenerative medicine is to limit the fibrous scar tissue formation while simultaneously promoting the intrinsic regenerative process, including SC performance, to achieve a morphofunctional recovery of a damaged tissue. In this context, expanding the knowledge of the biology of SCs is an essential requisite to offer cues for novel therapeutic approaches. To this aim, cutting‐edge imaging techniques like LSFM may offer insightful results concerning the visualization and location of SCs, both quiescent and activated. This can be particularly important, especially when considering the heterogeneity of the SC population and that in healthy skeletal muscle tissue, SCs are very few in number. In fact, SCs account for only 2%–10% of total myonuclei in adult muscles (from 2 × 10^5^ to 1 × 10^6^ cells/g muscle, depending on muscle and myofiber types) and in injured muscles, their percentage may increase up to ~10%–15% (Guilhot et al. 2024).

## 2 Materials and Methods

### 2.1 Animals

C57BL/6 (Charles River, Lecco, Italy) female mice (*n* = 11) were housed in the Laboratory Animal Facility (CeSAL, Centro Stabulazione Animali da Laboratorio, University of Florence, Florence, Italy) at 23°C± 1°C, fed with standard laboratory diet and water ad libitum, with a 12 h light/dark cycle. The animal study protocol was in accordance with the guidelines of the European Communities Council Directive 2010/63/UE and approved by the Institutional Animal Care Committee (University of Florence, Italy), subjected to the authorization of the Italian Ministry of Health (0DD9B.N.B9D/2024).

### 2.2 Ex Vivo Muscle Damage Induction

*Extensor digitorum longus* (EDL) muscles were isolated from mice (8/12 weeks, 25–30 g) formerly sacrificed by cervical dislocation. Once excised by cutting the tendons, one EDL muscle was used as a control (CTRL), whereas that of the contralateral limb was subjected to forced eccentric contraction (EC) in isometric condition, according to a previously published procedure with minor modifications (Squecco et al. 2021). Briefly, the EDL muscle was pinned on a sylgard‐coated chamber, applying a stretch of ~20% of the muscle resting length. In this isometric condition, the muscle was bathed for 1 min in the following contracting solution containing a high K^+^ concentration: 150 mM K‐glutamate, 2.0 mM MgCl_2_, 10 mM KOH, 10 mM TES, and 1.0 mM K_2_‐EGTA (Sigma‐Aldrich, Milan, Italy). This solution causes membrane depolarization over the mechanical threshold, followed by the contractile response. After each period in the contracting solution, the muscle relaxed for 4 min in a physiological solution (118 mM NaCl, 4.7 mM KCl, 1.2 mM MgSO_4_, 1.2 mM KH_2_PO_4_, 25 mM NaHCO_3_, 10 mM glucose, and 2.5 mM CaCl_2_) (pH 7.4 with NaOH, Sigma‐Aldrich). These contracting and relaxing cycles were repeated 10 times. After that, the muscles were processed for morphological analyses or subjected to electrophysiological recordings.

### 2.3 Hematoxylin and Eosin (H&E) Staining

CTRL and EC‐damaged muscles (*n* = 2 each) were fixed with 10% buffered formalin, dehydrated with a graded alcohol series, cleared in xylene, and then embedded in paraffin (*n* = 2 each). Paraffin‐embedded sections (5 μm thick) were deparaffinized, routinely stained with H&E, and observed under a light microscope (Nikon Eclipse E200, Nikon Europe B.V., The Netherlands, Europe) equipped with a Nikon Digital Sight DS U3 camera (Nikon).

### 2.4 Confocal Laser Scanning Microscopy (CLSM)

Paraffin embedded sections of CTRL and EC‐damaged muscles (at least 10 per muscle sample) were incubated with the fluorescent dye propidium iodide (1:100, for 2 min at room temperature‐RT‐Molecular Probes‐Thermo Fisher Scientific, Eugene, OR, USA) to label nuclei. Observations were performed under a confocal Leica TCS SP5 microscope (Leica Microsystems, Mannheim, Germany) equipped with a HeNe/Ar laser source for fluorescence measurements and differential interference contrast (DIC) optics by using a Leica Plan Apo 63×/1.43 NA oil‐immersion objective. Series of optical sections (1024 × 1024 pixels each; pixel size 204.3 nm) were taken through the depth of the samples at intervals of 0.4 μm (Z‐step size) and projected onto a single “extended focus” image.

### 2.5 Transmission Electron Microscopy (TEM)

For TEM analysis, CTRL and EC‐injured muscles (*n* = 2 each) were fixed with a 4% cacodylate‐buffered glutaraldehyde (pH 7.4) solution for 2 h, post‐fixed in 1% OsO4 in 0.1 M phosphate buffer (pH 7.4) for 1 h at RT, dehydrated in a graded acetone series, passed through propylene oxide, and embedded in Epon 812 (Sigma‐Aldrich). The ultrathin sections (60 nm thick) were contrasted with UranyLess stain and alkaline bismuth subnitrate (Electron Microscopy Sciences, Foster City, CA, USA) and then examined using a Jeol 1010 electron microscope (Jeol, Tokyo, Japan) at 80 kV equipped with a digital camera (Veleta, EMSIS GmbH, Münster, Germany).

### 2.6 Electrophysiological Recordings

Electrophysiological analysis was achieved on CTRL and EC‐injured EDL muscles (*n* = 2 each) as described in previous papers (Manetti et al. 2019; Squecco et al. 2021). Briefly, the employed setup (Axon Instruments, Burlingame, CA, USA) consisted of a digital‐to‐analog and analog‐to‐digital converter Digidata 1200 interface, an Axopatch 200B amplifier, and the software pCLAMP (version 6.02, Axon Instruments). The EDL muscles were pinned on the floor of the experimental chamber containing a Ringer‐Krebs solution (mM): 120 NaCl, 5 KCl, 2 CaCl_2_, 1 MgCl_2_, 5.5 HEPES, and 1 D‐glucose (Sigma‐Aldrich). Electrophysiological records were executed by introducing a microelectrode into a single skeletal muscle fiber of the CTRL and EC‐injured EDL muscles (*n* = 5 myofibers for each muscle). The microelectrodes were pulled from borosilicate glass capillaries (GC 100–7.5; Harvard Apparatus LTD) by using a vertical puller (Narishige PC‐10; Narishige International Inc., East Meadow, NY, USA) and then filled with an internal filling pipette solution containing (mM): 130 KCl, 10 NaH_2_PO_4_, 0.2 CaCl_2_, 1 EGTA, 5 MgATP, and 10 HEPES/KOH (pH 7.2) (Sigma‐Aldrich). The tip resistance was around 60 MΩ. The membrane passive properties of the skeletal muscle fibers (linear capacitance, Cm, and cell conductance, Gm) were recorded in voltage‐clamp mode, applying two 75‐ms‐long voltage step pulses to −80 and −60 mV (holding potential, HP = −70 mV). The specific membrane conductance (Gm/Cm) is an index of membrane permeability. Experiments were made at RT (22°C). The statistical analysis was performed by using Excel (Microsoft Office 2016, Microsoft corporation, Redmond, WA, USA) and Student's *T* test. Data are reported as mean ± standard deviation (SD). *p* < 0.05 has been considered statistically significant. n indicates the number of fibers investigated.

### 2.7 Light Sheet Fluorescence Microscopy

#### 2.7.1 Tissue Clearing and Staining

Muscle tissue clearing and staining was performed by adapting the iDISCO+ framework (Renier et al. 2016), a widely validated protocol for three‐dimensional imaging of thick samples.

Fixed muscles (5 CTRL and 5 EC) were washed in PBS (pH 7.6) for 30 min twice and then treated with increasing concentrations of ddH_2_O/Methanol (MeOH) (20%, 40%, 60%, 80%, and 100% twice) for the dehydration process. The incubation time was 30 min per step, and it was performed at RT. After the second wash in 100% MeOH, the samples were incubated overnight at RT in a solution composed of 66% (v/v) Dichloromethane (DCM) (270997—250 mL, Sigma Aldrich) and 33% (v/v) MeOH with shaking. The following day, samples were washed twice in 100% MeOH (1 h per wash, RT, shaking) and bleached overnight at 4°C in freshly prepared 5% hydrogen peroxide (H_2_O_2_) in MeOH.

For pseudo‐H&E staining, samples were washed twice in 100% MeOH and incubated overnight with 1:100,000 (w/v) Eosin Y (#HT110232, aqueous, 5% w/v, Sigma‐Aldrich), dissolved in 100% MeOH at RT with gentle shaking. On the fourth day, excess eosin was removed by hourly washes in 100% MeOH until the solution cleared. Subsequently, nuclei labeling was performed with 1:5000 (v/v) SYTOX Deep Red (Thermofisher, Milano, Italy) in 100% MeOH for 1 h at RT with shaking.

The last day, in order to remove the excess of SYTOX Deep Red, samples were firstly washed twice for 30 min each with 100% MeOH and then incubated in 66% DCM/33% MeOH for 1.5 h and washed in 100% DCM for 15 min twice, all at RT with gentle shaking. Lastly, refractive index matching (RI matching), a step that allows unhindered light transmission through both the sample during imaging and storage, was achieved by dibenzyl ether incubation (DBE) (#108014, Sigma Aldrich).

For immunostaining, after bleaching, samples were rehydrated by reversing the sequence of H_2_O/MeOH incubations starting from the 60% dilution and then washed in PBS and treated twice with 0.2% Triton X‐100 (Sigma Aldrich) in PBS (PBTx.2). Subsequently, permeabilization was performed at 37°C for 24 h (shaking) using a Permeabilization solution (0.2% (v/v) Triton X‐100, 20% (v/v) Dimethyl Sulfoxide (DMSO), 2.3% (w/v) Glycine (Sigma Aldrich) in PBS). The day after, samples were moved to *Blocking Solution* (0.2% (v/v) Triton X‐100, 0.01% (w/v) Sodium Azide (Sigma Aldrich), 0.2% (w/v) Gelatin Porcin Skin (Sigma Aldrich, G1890), and left at 37°C, with shaking for another 24 h). The immunolabeling was then executed incubating the samples respectively with the following primary antibodies: rabbit polyclonal anti‐Pax‐7 [#PA5‐68506; Invitrogen, Life Technologies, Grand Island, NY, USA; 1:500 (v/v)] rabbit polyclonal anti‐MyoD [#M‐318, sc‐760, Santa Cruz Biotechnology, Santa Cruz, CA; 1:200 (v/v)] Both antibodies were diluted in a buffer composed of 0.2% (v/v) Tween‐20, 0.001% w/v Heparin (# H5515‐100KU, Sigma‐Aldrich) in PBS (PBSTw‐H) for 72 h at 37°C with shaking. After washing for 24 h with PBSTw‐H, secondary antibody incubation was performed by moving the sample to 1:1000 (v/v) of the corresponding secondary antibody (Abcam, Cambridge, UK, goat anti‐rabbit Alexa Fluor 568, #ab175471) diluted in PBTw‐H for 24 h at 37°C with shaking. Another day of washing was performed with PBSTw‐H, and afterward, samples were dehydrated by reversing the sequence of ddH_2_O/MeOH incubations and stored overnight in 100% MeOH at RT with shaking. The last steps, from the staining procedure for nuclei labeling to storage, were conducted as described for the pseudo‐H&E staining.

#### 2.7.2 LSFM Imaging

Whole muscle imaging was performed with a custom LSFM apparatus described in detail in Laurino et al. (2023). The light sheet was generated in digital scanning mode using a galvanometric mirror (6220H, Cambridge Technology, Bedford, MA, USA); confocal detection was achieved by synchronizing the galvo scanner with the line read‐out of two different sCMOS cameras, one for each fluorescence channel (Orca Flash4.0, Hamamatsu Photonics, Shizuoka, Japan). Laser light was provided by diode and DPSS lasers (Cobolt, HÜBNER Photonics GmbH, Germany); an acousto‐optic tunable filter (AOTFnC‐400.650‐TN, AA Opto‐Electronic, France) was used to adjust laser intensity. For pseudo‐H&E imaging, 561 nm and 638 nm excitation lasers were used; for immunostaining, only the 561 nm excitation laser was used. The excitation objective was a 10×, 0.3 NA Plan Fluor from Nikon, while the detection objective was a 10×, 0.6 NA Plan Apochromat from Olympus. The whole sample was recorded using a cuvette containing DBE. The cuvette was mounted on a motorized *x*‐, *y*‐, *z*‐, ‐stage (M‐122.2DD and M‐116.DG, Physik Instrumente, Karlsruhe, Germany), which allowed free 3D motion and rotation. Stacks were acquired with a z‐step of 2 μm and an xy pixel size resulting from the setup configuration of 0.65 μm, covering a field of view for a single image tile of 1.3 mm × 1.3 mm. The optical resolution of the system is about 1.3 μm in *xy* and 8 μm in *z* (equivalent to the light sheet thickness), and was measured in a previous work (Di Giovanna et al. 2019). The microscope was controlled via custom‐written code (https://github.com/lens‐biophotonics/SPIMlab), which coordinated the galvo scanners, the rolling shutter, and the stack acquisition.

#### 2.7.3 LSFM Image Analysis

Each whole‐muscle dataset consists of multiple image stacks (tiles), arranged in a 2D lattice, spaced by 1 mm. Each stack covers the entire depth of the sample in *z*. A standard dataset consists of about 15 stacks, and each stack is usually 2048 × 2048 × 1600 voxels, tantamount to about 12.5 GB for each channel. The entire dataset is thus in the range of 150–200 GB (raw data). Image tiles were stitched together using ZetaStitcher (https://github.com/lens‐biophotonics/ZetaStitcher). To enable visualization of whole‐muscle images on a standard workstation, a downscaled version was also created (voxel size 5.2 × 5.2 × 5 μm^3^) with a typical size of 1.25 G. Pseudo‐H&E coloring was achieved, starting from eosin and SYTOX Deep Red fluorescence images, using the computational method described by Laurino et al. (2023). Image visualization was performed using FIJI (https://imagej.net/software/fiji/).

## 3 Results and Discussion

**FIGURE 1:**
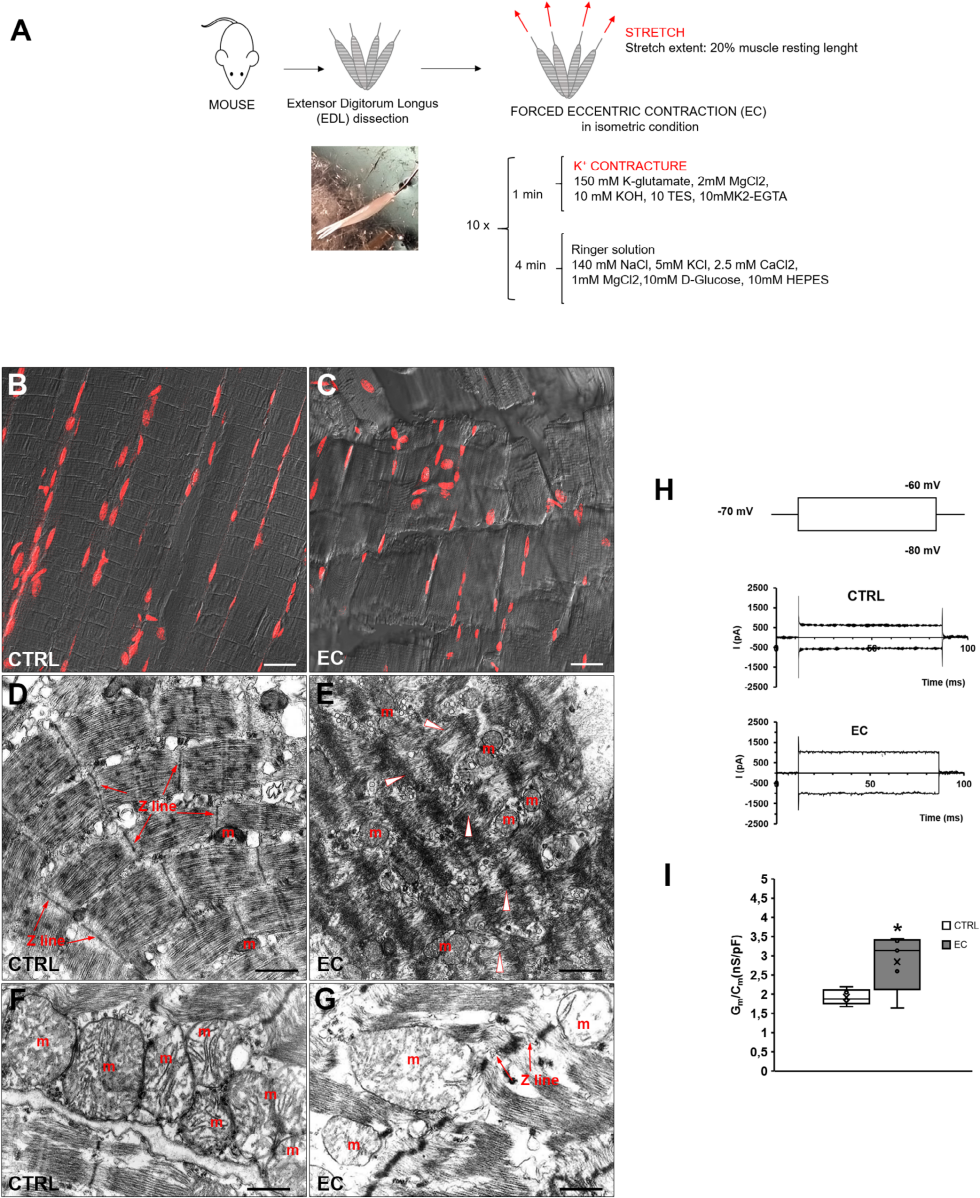
Ex vivo murine muscle (EDL) damage model validation. (A) Schematic representation of the experimental model. (B, C) Representative superimposed differential interference contrast (DIC, gray) and confocal laser scanning fluorescence (CLSM) images acquired simultaneously of muscle tissue sections of control (CTRL) and damaged EDL muscle (EC) showing myonuclei labeled with propidium iodide (red). Scale bar: 25 μm. (D, G) Ultrathin sections of CTRL and EC‐damaged muscles stained with UranyLess and bismuth subnitrate solutions and observed by transmission electron microscopy (TEM). m = mitochondria; arrowheads indicate sarcomere/myofilament disorganization. Scale bar: D, E, 1 μm; F, G, 300 nm. (H) Voltage‐step pulse protocol (upper panel) and representative passive current response obtained in a CTRL (middle panel) and in a EC EDL muscle fiber (lower panel). (I) Specific conductance (Gm/Cm) values estimated in the two conditions. *p < 0.05 (*n* = 5, Student's *T*‐test).

The proposed high resolution imaging method is applied on murine hindlimb skeletal muscles (EDL), one of which was kept as healthy muscle (CTRL) and the contralateral one was injured by means of EC (Figure 1A). The validity of this kind of ex vivo muscle injury model was first validated by means of morphological evaluations (CLSM and TEM) and electrophysiological functional analyses (Figure 1B–I). The results were essentially in accordance with our previous reports (Manetti et al. 2019; Sassoli et al. 2011; Squecco et al. 2021). Indeed, EC injured samples exhibited alteration of the myofiber structure, namely Z‐disc smearing/streaming and focal loss of myofilaments associated with their misalignment. Nuclei of EC myofibers appeared more rounded and distributed through sarcoplasm in comparison to elongated nuclei of control myofibers typically located at myofiber periphery (Figure 1B,C). Ultrastructural analysis highlighted an evident disorganization of the sarcomeres, the disruption of Z‐disc, and swollen abnormal mitochondria with disarranged or missing cristae (Figure 1D,E). Moreover, analysis of the sarcolemma passive properties confirmed functional anomalies of EC myofibers. The current responses evoked in EC muscle fibers used to show a leakage current with a larger amplitude than CTRL fibers (Figure 1H). Based on first Ohm's law (*V* = *IR*), being *V* = 10 mV in any case, this indicates a lower resistance and thus a higher conductance, Gm, in injured muscle fibers, suggesting a leakier membrane in EC muscle fibers. Indeed, this was confirmed by the specific conductance (Gm/Cm) analysis that showed statistically significant differences (*p* = 0.018) between values evaluated in CTRL and those in EC muscles (Figure 1I).

**FIGURE 2:**
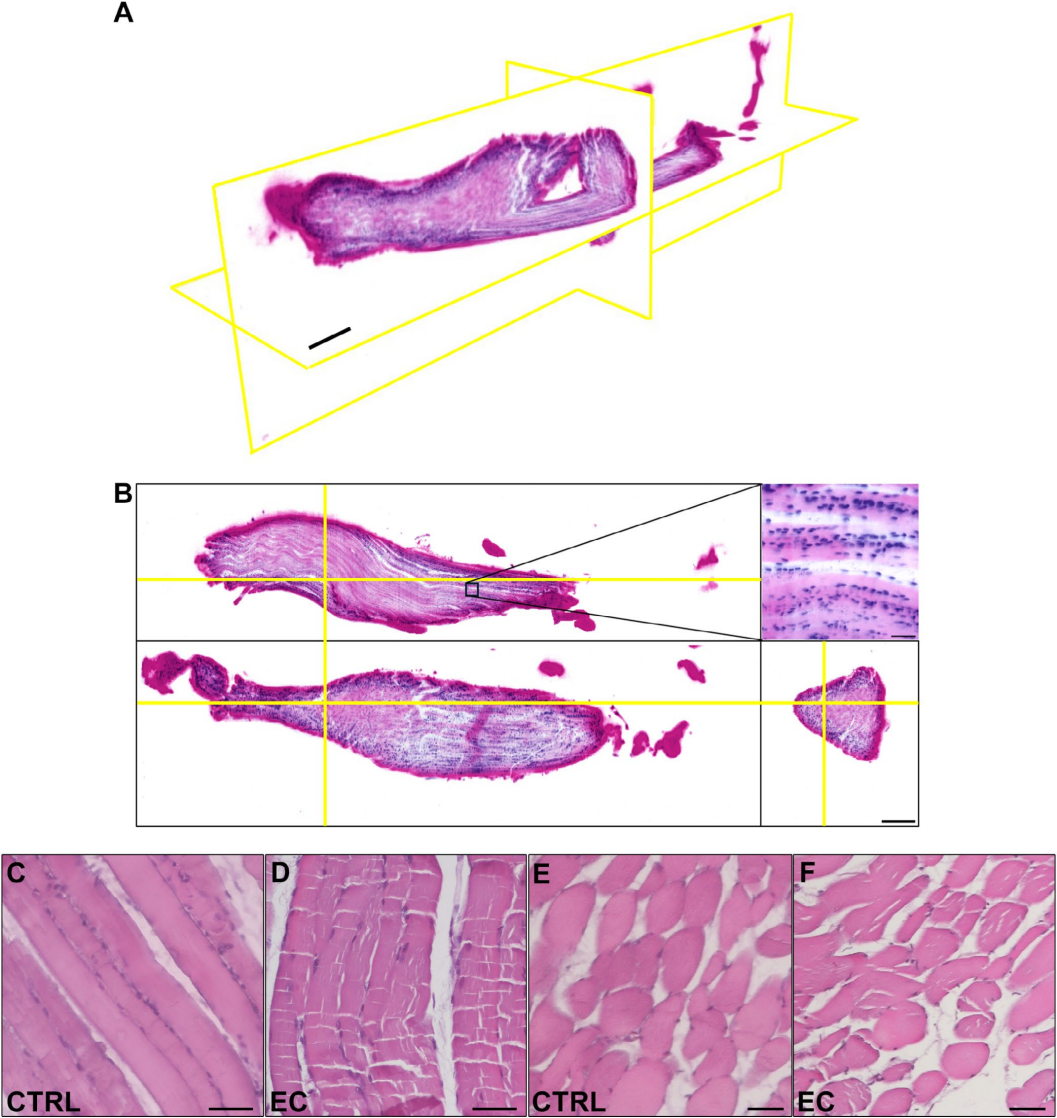
(A, B) 3D structural imaging of intact muscles by light sheet fluorescence microscopy (LSFM). (A) Volumetric reconstruction of an intact muscle sample (EC) imaged in fluorescence with eosin and SYTOX Deep Red, and rendered using false H&E colors. (B) Orthoslices extracted from the entire volume. Yellow lines identify the other two orthoslice in each image. The zoom‐in highlights that the resolution of the system is enough to distinguish single nuclei. Orthoslices and volumetric reconstruction are obtained from downsampled data, the zoom‐in from native resolution data. Scale bars in A and B: 500 μm (orthoslices), 50 μm (zoom‐ins). (C–F) Traditional H&E images showing 2D histology from (C, D) longitudinal and (E, F) transverse sections of the indicated muscle samples. Scale bar: 100 μm.

For the first time we explored the use of LSFM and tissue clearing to analyze the localization and activation of SCs in the full volumetric structure of entire skeletal muscles. First, we labeled the sample using eosin and a nucleic acid marker (SYTOX Deep Red), obtaining a fluorescence analog of standard histological H&E staining, as described in Laurino et al. (2023). By applying a simple mathematical transformation to the original fluorescence data, it is possible to render the images in the H&E color space (Figure 2). The entire volume can be visualized in 3D (Figure 2A), and the user can extract virtual slices along any direction of interest (Figure 2B). Essentially, the high resolution of our custom LSFM apparatus enables us to clearly visualize single nuclei even in the tightly packed muscular environment (Figure 2B, inset). Our pseudo‐H&E images are similar to true H&E slides cut along longitudinal (Figure 2C,D) and transversal directions (Figure 2E,F), that are shown in the bottom part of the figure for a more direct comparison of the two methodologies. However, a more accurate inspection of these data shows that the penetration of dyes is not homogeneous, as evident from the darker coloring of tissue borders. This is in line with previous reports on cardiac muscular tissues, where homogenous staining was shown to be challenging (Olianti et al. 2022). Nonetheless, tissue coloration is also effective in core regions: visual inspection of the tissue can thus be achieved by locally adjusting the contrast.

**FIGURE 3:**
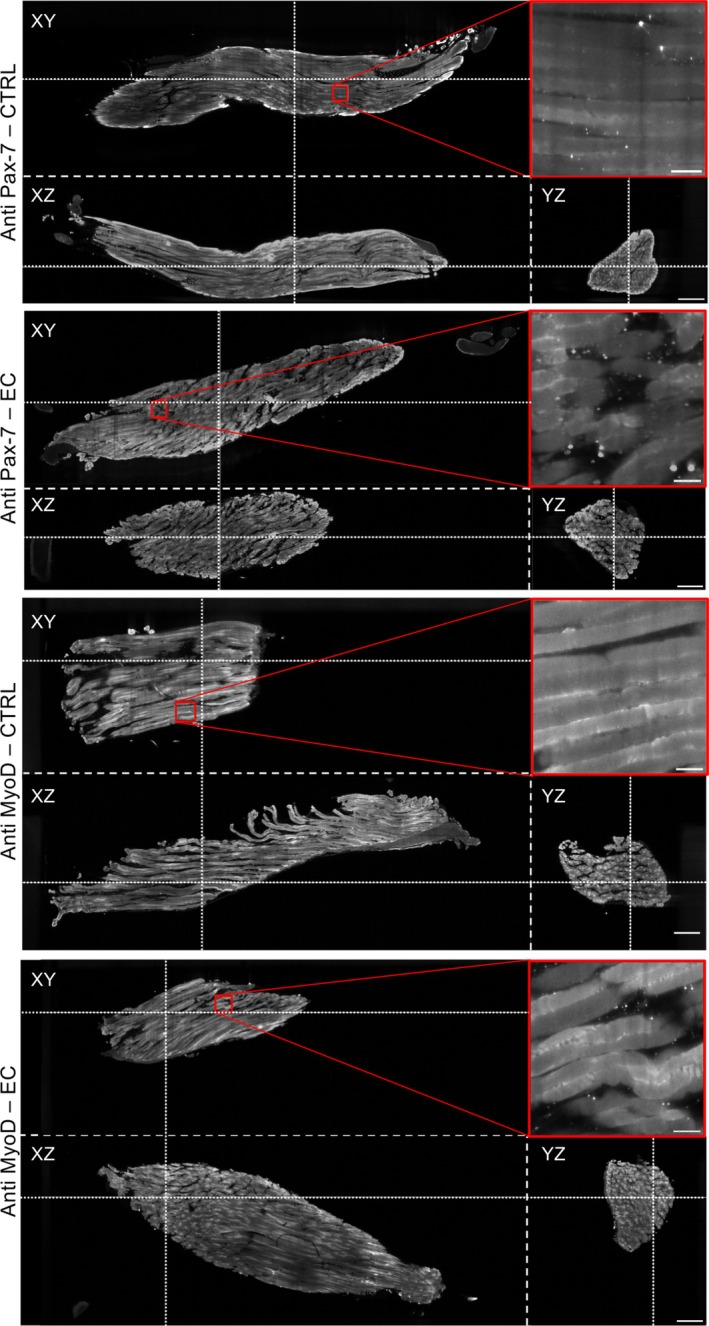
3D immunostaining of intact muscles; both control (CTRL) and damaged muscle (EC) were analyzed. Orthoslices extracted from the entire volume of muscles were stained against Pax‐7 (top panels) and MyoD (bottom panels). Dotted lines identify the other two orthoslices in each image. Zoom‐ins highlight the resolution of the system needed to identify small satellite cells. Orthoslices are obtained from downsampled data; the zoom‐in is from native resolution data. Scale bars: 500 μm (orthoslices), 50 μm (zoom‐ins).

In order to identify SCs in cleared tissue preparations, we performed immunostaining against Pax‐7 or MyoD (Figure 3). As expected, resting SCs—targeted by the anti‐Pax‐7 antibody—were identified in both CTRL and EC samples (Figure 3, top panels). On the other hand, activated SCs—which are labeled by anti‐MyoD immunohistochemistry—are not visible in CTRL muscles while they become apparent in EC‐damaged samples (Figure 3, bottom panels). Here, we were not able to perform a simultaneous double immunostaining against both targets, as the antibodies used were both raised in the same host species (rabbit). Double immunolabeling might certainly provide a more comprehensive scenario and more insightful data concerning the muscle regenerative response to injury in terms of behavior, activation, myogenic state, and spatial distribution of the “different” quiescent and myogenic activated SCs. Indeed, quiescent SCs are characterized by the expression of Pax‐7, which is regarded as the canonical specific marker of SCs necessary for their survival and functionality, and of the myogenic transcription factor Myf5, detectable in ~90% of quiescent SCs. In this dormant state, SCs did not express the MRFs, such as MyoD or myogenin. In injured muscles, in response to signals coming mainly from the injured microenvironment, SCs exit from the quiescent state, re‐enter the cell cycle, and closely recapitulate the steps of the embryonic and fetal myogenesis program. In particular, at first, SCs undergo mitotic division, giving rise to a progeny of proliferating myoblasts co‐expressing Pax7, Myf5, and MyoD. Subsequently, these cells downregulate Pax7, express the late myogenic marker, namely myogenin and MRF4/Myf6 expression, and differentiate into skeletal myocytes that fuse with each other to form new syncytial contractile myofibers or fuse with injured myofibers to repair the tissue damage. Moreover, a small percentage of SCs considered the “true” stem cells self‐renew to ensure the replenishment of the basal pool of resident SCs recruitable for future demand (Guilhot et al. 2024).

The indirect immunofluorescence labeling performed in this study requires the combination of primary antibodies obtained in different host species and preferentially not from the species of the tested tissue in order to avoid cross reactions. Unfortunately, it is not easy to commercially find such tools in the suitable combination. To overcome this criticism, future investigations could be planned with direct immunolabeling techniques by using fluorescent primary antibodies.

In addition to the immunostaining, the images show a distinct autofluorescence, enabling visualization of the underlying tissue structure. This is also helpful to identify where the SCs are located with respect to the muscular fibers. Interestingly, data obtained through immunostaining show better homogeneity than those performed with pseudo‐H&E, suggesting that the mechanisms that affect dye diffusion are not dictated by molecular size but likely by affinity or electrostatic interactions. More generally, it would be important in the future also to evaluate different clearing and staining methods to improve penetration of different dyes (Vermoortele et al. 2024).

These first data on volumetric imaging of SCs in intact skeletal muscles represent a methodological proof‐of‐principle study that paves the way for new biology and, potentially, new medicine. Indeed, the possibility of visualizing the entire sample enables more reliable cell counting, especially when dealing with rare objects such as SCs (Liu et al. 2021). In the translational perspective of regenerative medicine, it is urgent to estimate, localize, and eventually enhance the activity of all SCs resident in the skeletal muscular tissue. Besides the case study of SCs, analysis of skeletal muscles in 3D can simplify the laboratory flow, as there is no need to decide in advance the orientation of the specimen or the location of the slice under analysis. Computational reconstructions obtained with 3D optical imaging can be analyzed by a pathologist without any specific training in volumetric microscopy, paving the way for new biomedical applications in clinical pathology. Since LSFM provides a full digital twin of the original sample, the researcher can decide in the aftermath which areas and planes of the sample need to be visualized.

## 4 Conclusion

In this letter, we demonstrate the feasibility of a novel procedure to analyze the localization of quiescent and activated SCs, imaging the entire skeletal muscle in 3D with cellular resolution. This protocol, based on tissue clearing, immunostaining, and LSFM system analysis, might be used in the field of skeletal muscle regenerative medicine to study the intrinsic muscle regeneration process in a more comprehensive way, to eventually identify any pathological alterations (e.g., in neuromuscular diseases) and optimize regenerative therapies or targeted pharmacological treatments.

## Author Contributions

**Rachele Garella:** methodology, visualization, validation, investigation, writing – review and editing, formal analysis. **Elisa Imbimbo:** funding acquisition, methodology, investigation, validation, writing – review and editing, formal analysis, data curation. **Francesco Palmieri:** methodology, investigation, validation, data curation, writing – review and editing. **Alessia Tani:** methodology, validation, writing – review and editing, investigation, data curation. **Martina Parigi:** methodology, validation, writing – review and editing, investigation, data curation. **Flaminia Chellini:** methodology, validation, writing – review and editing, investigation, data curation. **Alessandra La Contana:** methodology, validation, writing – review and editing, investigation, data curation. **Monica Mattioli Belmonte:** writing – review and editing, funding acquisition. **Aurora Longhin:** methodology, validation, investigation, writing – review and editing, data curation. **Ludovico Silvestri:** conceptualization, methodology, resources, data curation, validation, investigation, writing – review and editing, writing – original draft, visualization, project administration, funding acquisition, supervision. **Chiara Sassoli:** conceptualization, methodology, validation, data curation, investigation, resources, visualization, writing – review and editing, writing – original draft, funding acquisition, formal analysis, supervision. **Roberta Squecco:** data curation, supervision, resources, formal analysis, writing – review and editing, visualization, validation, methodology, writing – original draft, funding acquisition, investigation, conceptualization.

## Conflicts of Interest

The authors declare no conflicts of interest.

## Data Availability

The data that support the findings of this study are available from the corresponding author upon reasonable request.
